# Anti-tuberculosis drug-induced hepatotoxicity in people living with HIV

**DOI:** 10.2478/abm-2026-0016

**Published:** 2026-06-30

**Authors:** Editorial Office of Asian Biomedicine

**Affiliations:** Faculty of Medicine, Chulalongkorn University, Bangkok 10330, Thailand

Tuberculosis (TB), caused by *Mycobacterium tuberculosis*, remains a major global public health challenge. Its burden is driven by poverty, limited healthcare infrastructure, delayed diagnosis, and the growing prevalence of drug-resistant strains, as well as its syndemic interaction with human immunodeficiency virus (HIV). According to the World Health Organization, approximately 1.23 million people died from TB in 2024, including 150,000 individuals living with HIV [1]. A substantial proportion of these deaths is attributable to multidrug-resistant TB, for which access to effective treatment remains inadequate in many settings.

Despite TB being both preventable and curable, significant challenges persist. Advances in TB management—including optimized pharmacological regimens, improved diagnostics, and adjunctive therapies—have enhanced treatment outcomes for both drug-sensitive and drug-resistant TB. However, anti-TB drug-induced hepatotoxicity (DIH) continues to be a major complication, particularly in vulnerable populations such as people living with HIV (PLWH) [2].

The incidence of antituberculosis liver injury (ATLI) varies across geographic regions and patient populations. A pooled analysis estimated an overall incidence of 11.5% (95% CI: 10.10%–12.97%), with an increasing trend over time. Higher rates have been observed among patients receiving first-line anti-TB regimens (13.66%), those in South America (18.16%), and individuals co-infected with hepatitis B or C viruses (39.19%).

Importantly, the incidence of ATLI is significantly higher in PLWH, often necessitating treatment interruption and contributing to increased morbidity and mortality [3]. Several mechanisms may explain this elevated risk, including HIV-related immune dysregulation, direct hepatic effects of the virus, and hepatotoxicity associated with antiretroviral therapy (ART). The combined exposure to anti-TB drugs and ART may further exacerbate liver injury [4]. In addition, malnutrition remains an important and often under-recognized risk factor for ATLI [5].

In this issue, Atuel et al. [6] reported treatment outcomes among patients who developed DIH during anti-TB therapy. Notably, their findings suggest that treatment success rates remain high despite the occurrence of hepatotoxicity, and that anti-TB drug-induced hepatitis does not significantly compromise overall TB treatment outcomes.

Early initiation of ART—either before or during TB treatment—is essential to improve survival and reduce HIV-related complications, although careful monitoring for hepatotoxicity is required [7]. Therefore, national TB and HIV programs should consider expanding ART coverage among individuals co-infected with TB and HIV to better control this dual epidemic. Particular attention should be given to high-risk groups, including patients with hepatitis C co-infection, undernutrition, and other predisposing factors.
